# Upper Extremity Deep Vein Thrombosis and Asymptomatic Vein Occlusion in Patients With Transvenous Leads: A Systematic Review and Meta-Analysis

**DOI:** 10.3389/fcvm.2021.698336

**Published:** 2021-08-18

**Authors:** Daniël Duijzer, Maria A. de Winter, Mathilde Nijkeuter, Anton E. Tuinenburg, Jan Westerink

**Affiliations:** ^1^Department of Vascular Medicine, University Medical Center Utrecht, Utrecht, Netherlands; ^2^Department of Acute Internal Medicine, University Medical Center Utrecht, Utrecht, Netherlands; ^3^Department of Cardiology, University Medical Center Utrecht, Utrecht, Netherlands

**Keywords:** deep vein thrombosis, cardiac device therapy, transvenous leads, epidemiology, systematic review and meta-analysis

## Abstract

**Aims:** The presence of transvenous leads for cardiac device therapy may increase the risk of venous thromboembolisms. The epidemiology of these complications has not yet been determined systematically. Therefore, this study aims to determine (I) the incidence of symptomatic upper extremity deep vein thrombosis (UEDVT) and (II) the prevalence of asymptomatic upper extremity vein occlusion in patients with transvenous leads, both after the initial 2 months following lead implantation.

**Methods:** PubMed, EMBASE, and Cochrane Library were searched until March 31, 2020 to identify studies reporting incidence of UEDVT and prevalence of asymptomatic vein occlusion after the initial 2 months after implantation in adult patients with transvenous leads. Incidence per 100 patient years of follow-up (PY) and proportions (%) were calculated to derive pooled estimates of incidence and prevalence.

**Results:** Search and selection yielded 20 and 24 studies reporting on UEDVT and asymptomatic vein occlusion, respectively. The overall pooled incidence of UEDVT was 0.9 (95% CI 0.5–1.4) per 100PY after 2 months after lead implantation. High statistical heterogeneity was present among studies (I^2^ = 82.4%; *P* = < 0.001) and only three studies considered to be at low risk of bias. The overall pooled prevalence of asymptomatic upper extremity vein occlusion was 8.6% (95% CI 6.0–11.5) with high heterogeneity (I^2^ = 81.4%; *P* = <0.001). Meta-regression analysis showed more leads to be associated with a higher risk of UEDVT.

**Conclusion:** Transvenous leads are an important risk factor for symptomatic UEDVT, which may occur up to multiple years after initial lead implantation. Existing data on UEDVT after lead implantation is mostly of poor quality, which emphasizes the need for high quality prospective research. Asymptomatic vein occlusion is present in a substantial proportion of patients and may complicate any future lead addition.

**Clinical Trial Registration:** (URL: https://www.crd.york.ac.uk/prospero/display_record.php?ID=CRD42020178136, Identifier: PROSPERO 2020 CRD42020178136).

## Introduction

Pacemakers and implantable cardioverter-defibrillators (ICD) are commonly used to respectively, control and restore heart rhythm. The European Society of Cardiology reported over 500,000 pacemakers and ~85,000 ICD implantations in 2013 and the prevalence of cardiac devices is likely to increase (1–3). For over 60 years, transvenous leads have been used in cardiac device therapy. Although, other manners of internal pacing and defibrillation have been developed, cardiac device therapy involving transvenous leads remains common practice.

The intravascular presence of leads comes with the risk of various complications. A long-known complication is the stenosis of the deep veins of the upper extremity, in which venous stasis and activation of the coagulation cascade–and the subsequent formation of a thrombus–play significant roles (4). Acute thrombosis in these veins may cause signs and symptoms of venous congestion–including oedema, pain, and fatigue–in the arm; a condition which is referred to as upper extremity deep vein thrombosis (UEDVT). In addition, venous thrombosis comes with the short term risk of an acute pulmonary embolism (5). On the long term, a post-thrombotic syndrome of the arm might develop (6). However, stenosis and even total occlusion of these deep veins often remains asymptomatic when a sufficient collateral vein system is present. Yet, an asymptomatic occlusion becomes clinically relevant when leads have to be added, complicating the procedure.

Previous studies found that UEDVT occurred in 0.2–0.7% after ICD implantation and replacement in the early postoperative period (7) and that venous thromboembolism (VTE) occurred in 0.5% of the patients within the first month after pacemaker implantation (8). Thrombotic complications occurring in the first months after lead implantation may be attributed to the surgical intervention, whereas, those occurring after the postoperative period might be provoked by the transvenous leads themselves. Since the pathogenesis of the thrombotic complications during and after the first postoperative months is likely to be different, the epidemiology might diverge with increasing time after lead implantation.

The precise epidemiology of non-surgery related thrombotic complications in patients with transvenous leads have to date not been reviewed systematically (4, 9–11). Determination of incidences and prevalence will give insight into the burden of thrombotic complications and helps to understand the association between transvenous leads and UEDVT and asymptomatic vein occlusion. Therefore, we aim to determine the incidence of UEDVT and prevalence of asymptomatic upper extremity vein occlusion in patients with transvenous leads for cardiac device therapy after the postoperative period by performing a systematic review and meta-analysis.

## Methods

This study was conducted in accordance with PRISMA and MOOSE-guidelines (12, 13). A protocol was registered in PROSPERO prior to conducting the study (registration ID: CRD42020178136).

### Data Sources and Search Strategy

An electronic search in PubMed, EMBASE and Cochrane Library was performed from inception until 31 March 2020. The search strategy consisted of free and controlled terms for cardiac device therapy and thromboembolic complications (Supplementary Data 1) and was constructed in cooperation with a librarian. The search was limited to publications in Dutch, English, French, and German. Reference lists of previous reviews of the subject as well as the included articles were hand-searched to identify additional eligible studies. Clinicaltrials.gov was searched for relevant ongoing studies.

Following retrieval of the search results, duplicates were removed (Mendeley version 1.19.4) and two reviewers (DD, MW) screened the title and abstract of the remaining records independently (Rayyan QCRI) (14). Relevant articles and articles of which eligibility could not be assessed properly were selected for full-text assessment. Disagreement whether or not to include a study after full-text assessment was resolved through debate with a third reviewer (JW) in order to reach consensus.

### Eligibility Criteria

Observational studies (cross-sectional, prospective, and retrospective cohort studies) and randomized controlled trials with a full-text article available were eligible for inclusion. Studies had to report on the incidence of UEDVT and/or prevalence of asymptomatic upper extremity vein occlusion in adult patients with active and/or abandoned leads for cardiac device therapy. Authors were contacted *via* email if additional information was required to assess eligibility. Studies were excluded if patients received temporary or transfemoral pacing, underwent haemodialysis, had a Fontan circulation; when the study focussed on perioperative management or lead extraction, and when postoperative follow-up of venous complications was restricted to 2 months or less. Finally, case reports, case series, post-mortem series as well as reviews were excluded.

### Data Extraction and Quality Assessment

One researcher (DD) extracted the data from the included studies using a predefined data extraction form (Supplementary Data 2). Adequate extraction of data was verified by a second researcher (MW) and disagreement was resolved by consensus through debate with a third reviewer (JW). In case of population duplicates, we included the most recent study or the one that reported most completely on the outcome of interest. Authors were contacted *via* email for additional data where necessary. Data was extracted on: first author; year of publication; study characteristics (country, design, aims, in- and exclusion criteria, sample size, follow-up duration, type of population); population characteristics (age, sex, predisposing factors for thrombosis, anticoagulant treatment, comorbidities, indication for and type of cardiac device, number of leads); definition and assessment of outcomes; incidence of UEDVT, follow-up duration at UEDVT and prevalence of asymptomatic vein occlusion.

Methodological quality and risk of bias of included studies was assessed by two reviewers (DD, MW) independently using the risk of bias tool of Hoy et al. (15), which is specifically developed for prevalence studies. With this tool, 10 items−4 on external and 6 on internal validity–are judged to be either at low or high risk of bias for each study. Item 9: *Was the length of the shortest prevalence period for the parameter of interest appropriate?* was considered inapplicable and was therefore omitted in the present study. A summary score of 0–1, 2–3, and 4–9 points represented low, moderate and high risk of bias, respectively. Complete risk of bias assessment of the studies included in the analysis can be found in Supplementary Data 3.

### Definitions and Outcomes

UEDVT was defined as any new thrombotic event with symptoms of venous congestion in the upper extremity occurring more than 2 months after transvenous lead implantation. The implantation procedure was considered a minor transient risk factor for VTE (16). Hence, all UEDVT occurring within 2 months after the implantation procedure were attributed to the procedure rather than the presence of transvenous leads and these cases were therefore excluded. Events of which timing in relation to the implantation could not be confirmed were included in the overall analysis and sensitivity analysis was conducted afterwards. Asymptomatic vein occlusion was defined as total occlusion of one or more deep veins of the upper extremity ascertained by venography or ultrasound without the presence of clinical symptoms of venous congestion. Visible superficial collateral vein formation by itself was not considered a symptom in both definitions. Primary outcomes were incidence rate per 100 patient years of follow-up (PY) for UEDVT and prevalence (%) for asymptomatic vein occlusion.

### Statistical Analysis

To compare the incidence of UEDVT across studies, we calculated incidence rates expressed as events per 100 PY, using the number of events and total follow-up time of the study population. Total PY for studies with cases confirmed to have occurred ≥2 months postoperatively or no cases were subtracted by 2 months per patient. For studies reporting median with interquartile range or total range, mean (μ) and standard deviation (SD) were estimated (17, 18). Prevalence of asymptomatic vein occlusion was expressed as proportion (%). Given the binary character of the data, the variance stabilizing double arcsine transformation was applied to calculate both incidence rate and prevalence (19).

Statistical heterogeneity was assessed using the Cochran's Q test (α < 0.1) and I^2^ statistic. The Cochran Q test tests whether there is significant heterogeneity among the reported effect sizes. The I^2^ statistic provides a quantitative estimate of the variability across studies; values of 0–25, 25–75, and 75–100% were considered to represent low, medium and high heterogeneity, respectively (20). Sensitivity analyses were conducted based on region; study design; risk of bias categories; assessed type of cardiac device; exclusion of patients with previous venous anomalies; whether number of events within 2 months postoperatively was unknown (UEDVT); patients with abandoned leads were subject of study (UEDVT); venography was used for ascertainment of outcome in all patients (asymptomatic vein occlusion).

To explore the source of heterogeneity, univariate random effects meta-regression analyses were conducted with incidence and prevalence as outcome in separate analyses and the following continuous variables as dependent variables: publication year; mean age; proportion of males; proportion of patients receiving anticoagulant therapy; mean number of leads per patient; and for the prevalence of asymptomatic vein occlusion mean follow-up time as well.

The DerSimonian and Laird random-effects model was applied because of the expected heterogeneity of studies included in the analysis (21). Potential publication bias was assessed by visual inspection of funnel plots and Egger's regression test (22). All statistical analyses were performed with the R Statistical Software (version 4.0.0; https://www.r-project.org/) using the metafor package (23). *P*-values <0.05 were considered statistically significant.

## Results

### Study Identification and Selection

**Figure 1:**
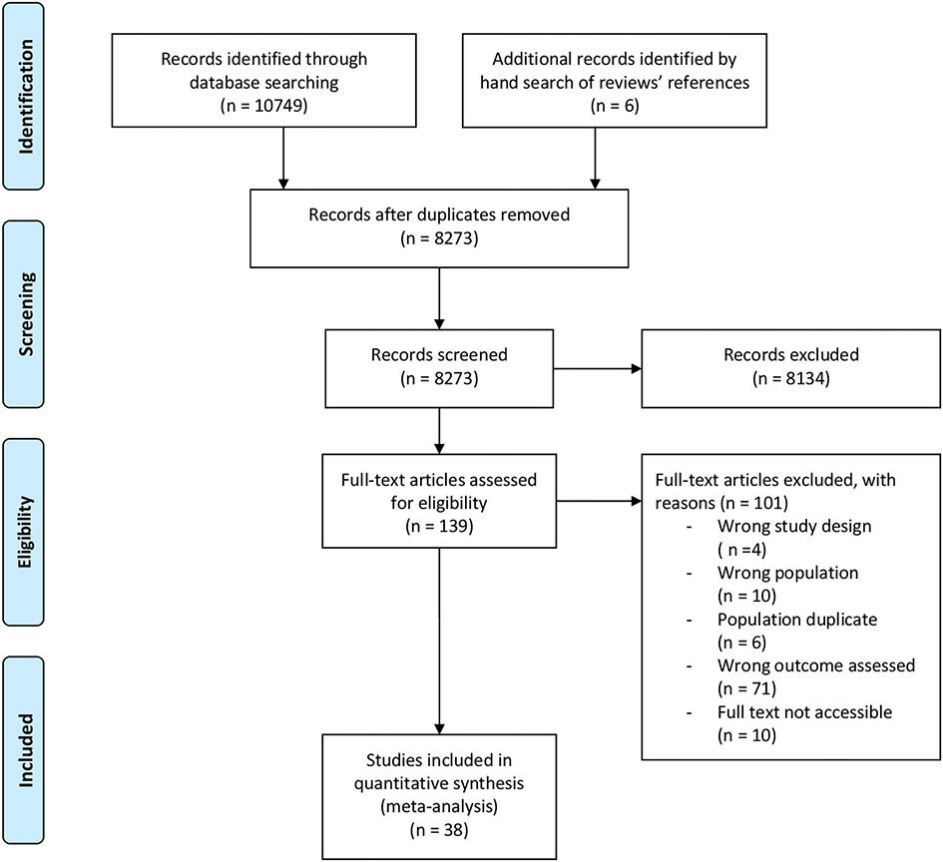
Flow diagram of study selection process for meta-analysis.

Out of 8,273 reports retrieved by the search strategy, 139 were in accordance with the eligibility criteria based on title and abstract. At full text assessment of these reports, 101 were excluded (Figure 1). Among the 38 included studies in the present systematic review, 14 studies report incidence of UEDVT (24–37), 12 report prevalence of asymptomatic vein occlusion (38–49) and 12 report both outcomes (50–61). No additional eligible reports were identified from a hand search of the included articles' references.

### Symptomatic UEDVT

Out of 26 studies reporting incidence of UEDVT, 6 were not included in the meta-analysis due to unavailable total person years of follow-up (26–29, 52, 53). Of the remaining 20 studies (median year of publication 2007) (24, 25, 30–37, 50, 51, 54–61), one study reported on two separate populations of patients with transvenous leads; one population having leads in use and the other having abandoned leads *in situ*. Patient populations were recruited from Europe (*n* = 9), Asia (*n* = 5), the Middle East (*n* = 3), the USA (*n* = 2), and Brazil (*n* = 2). Two studies reported on a substantial larger cohort of patients (*n* = 903, PY = 3,695; *n* = 6,256, PY = 29,195) (30, 35) than the other studies, which described populations ranging from 21 to 202 patients (range of PY 17–1,323). Relevant characteristics of individual studies are presented in more detail in Supplementary Table 4.

**Figure 2:**
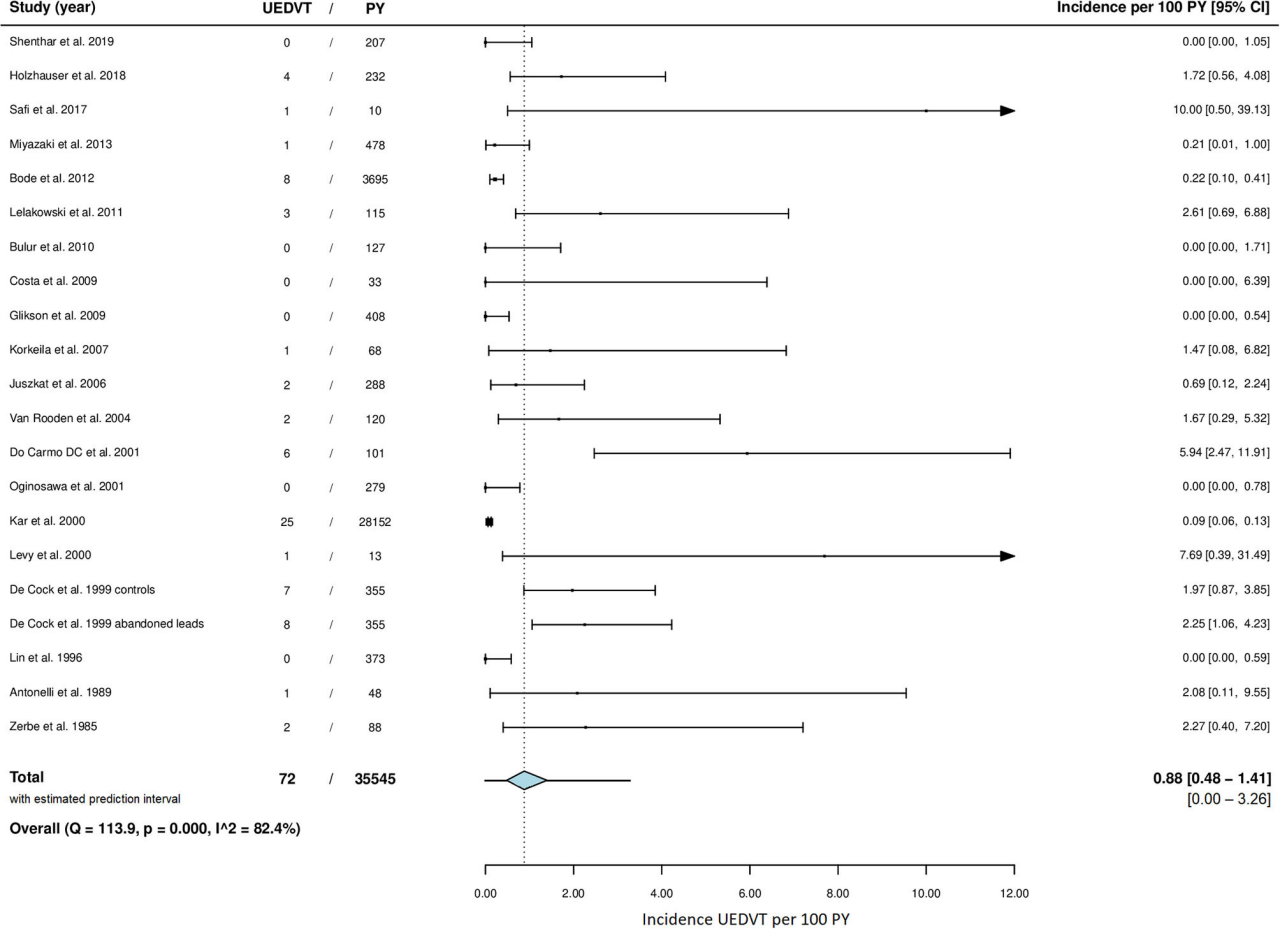
Meta-analysis of incidence rate of UEDVT. CI, confidence interval; PY, patient years; UEDVT, upper extremity deep vein thrombosis.

**Figure 3:**
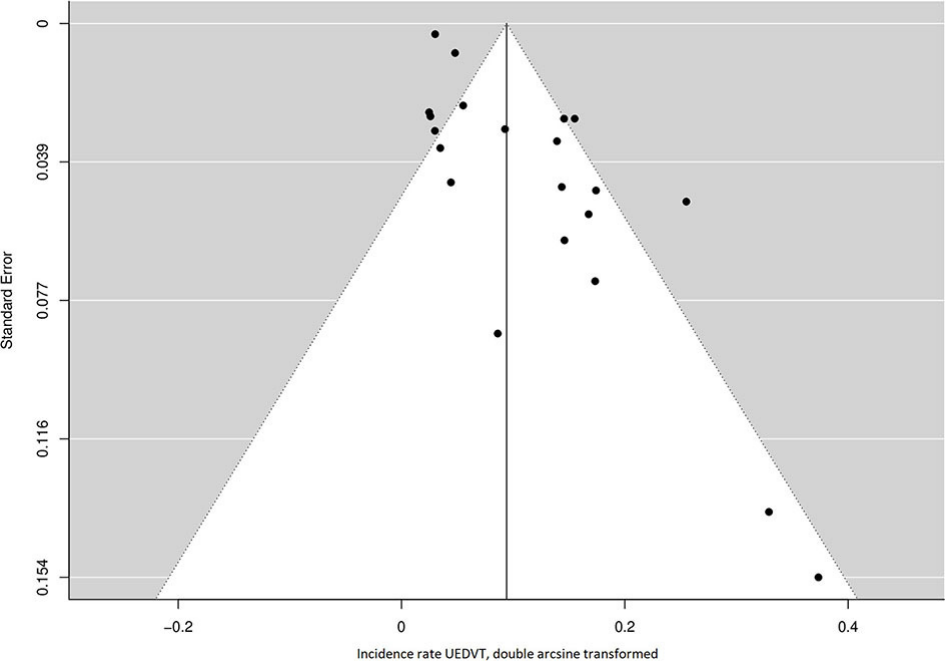
Funnel plot of the included studies for the incidence rate of UEDVT. UEDVT, upper extremity deep vein thrombosis.

A total of 72 cases of symptomatic UEDVT were reported in 8,671 patients followed for a total of 36,774 years, of which 35545 PY more than 2 months after lead implantation. Forty-two cases (58%) were confirmed to have occurred at 2 months or more post device implantation. The incidence rate of symptomatic UEDVT ranged from 0.0 to 10.0 cases per 100 years of follow-up across studies. The overall pooled incidence rate was 0.9 (95% CI 0.5–1.4) UEDVT per 100 PY, with high statistical heterogeneity among studies (I^2^ = 82.4%; *P* = <0.001) (Figure 2). Both the funnel plot, which was shaped asymmetrically (Figure 3), and Egger's test (*P* = <0.001) indicated publication bias.

UEDVT occurred at a median of 11 months (range 2–48) after lead implantation for the smaller studies (19 cases) and at a mean of 26 months postoperatively in the cohort described by Kar et al. (35).

Sensitivity analysis (Table 1) showed a pooled incidence rate of 0.6 (95% CI: 0.0–1.7) UEDVT per 100 PY for prospective studies and an incidence rate 0.2 (0.0–0.9) for retrospective studies. Including studies considered to be at low risk of bias only resulted in a pooled incidence rate of 1.3 (0.3–2.9) per 100 PY and addition of the studies regarded to be at moderate risk of bias resulted in an incidence rate of 0.1 (0.0–0.5) per 100 PY. Forming subgroups based on the included population resulted in an incidence rate of 0.7 (0.0–2.9) for patients with abandoned leads and 0.4 (0.0–1.4) for pacemaker only studies. The pooled incidence rate of the studies from which all cases of UEDVT were confirmed to have occurred later than 2 months postoperatively was 0.1 (0.0–0.5). A separate analysis with exclusion of the two largest study populations was performed (Supplementary Table 6), since these studies comprised 89% of the total included studies' PY (30, 35). A total of 39 cases of UEDVT remained in 3,698 PY of follow up with a pooled incidence rate of 1.2 (0.6–2.0) per 100 PY. Studies with inclusion of ICD patients only and studies from Asia had a significant lower incidence of UEDVT.

**Table 1:** Subgroup analysis for the incidence of symptomatic UEDVT.

**Subgroups**	**Populations (*n*)**	**Cases (*n*)**	**PY (*n*)**	**UEDVT per 100 PY % (95% CI)**	**Heterogeneity**	
					**I^**2**^**	**Q test's *P***
**Overall**						
	21	72	35,545	0.9 (0.5–1.4)	82.4	<0.001
**World part**						
Europe	9	34	5,097	1.1 (0.2–2.6)	81.0	<0.001
Asia	5	26	29,489	0.0 (0.0–0.0)	0.0	0.873
Middle East	3	2	185	1.5 (0.0–8.1)	65.3	0.056
USA	2	4	640	0.1 (0.0–3.0)	87.1	0.005
Brazil	2	6	134	2.7 (0.0–11.1)	65.2	0.090
**Design**						
Prospective	15	55	30,391	0.6 (0.0–1.7)	84.8	<0.001
Retrospective	5	17	4,781	0.2 (0.0–0.9)	69.9	0.010
**Risk of bias**						
Low	3	7	420	1.3 (0.3–2.9)	0.0	0.994
Low + moderate	11	20	5,648	0.1 (0.0–0.5)	60.1	0.005
**Cardiac device**						
Pacemaker only	12	47	30,146	0.4 (0.0–1.4)	83.6	<0.001
ICD only	2	1	886	0.0 (0.0–0.1)	0.0	0.336
**Population**						
Follow-up after device implantation	17	58	34462	0.2 (0.0–0.6)	79.6	<0.001
Abandoned leads	4	14	1,083	0.7 (0.0–2.9)	81.9	0.001
**Timing of events**						
All UEDVT ≥2 months post-op	15	42	30,938	0.1 (0.0–0.5)	72.9	<0.001
no. UEDVT <2 months post-op unknown	6	30	4,607	1.2 (0.0–3.6)	87.8	<0.001
**Patients with venous anomalies excluded**						
Yes	4	6	452	1.2 (0.0–4.5)	70.1	0.018
No or not stated	17	66	35,093	0.2 (0.0–0.7)	83.2	<0.001

*PY, person years of follow-up; UEDVT, symptomatic upper extremity deep vein thrombosis; CI, confidence interval*.

Univariate meta-regression analyses showed no association with publication year, age, sex, proportion of patients on anticoagulation. However, a higher mean number of leads per patient was significantly associated with a higher incidence rate of UEDVT (*P* 0.002) (**Table 3**).

### Asymptomatic Upper Extremity Vein Occlusion

Median publication year of the 24 studies included for prevalence of asymptomatic vein occlusion was 2002 (38–61). Studies were conducted in Europe (*n* = 8), Asia (*n* = 6), the Middle East (*n* = 5), the USA (*n* = 3), and Brazil (*n* = 2). The sample size of the studies ranged from 20 to 227 participants and patients were assessed at a median of 3.8 years after transvenous lead placement. Characteristics of individual studies are displayed in Supplementary Table 5.

**Figure 4:**
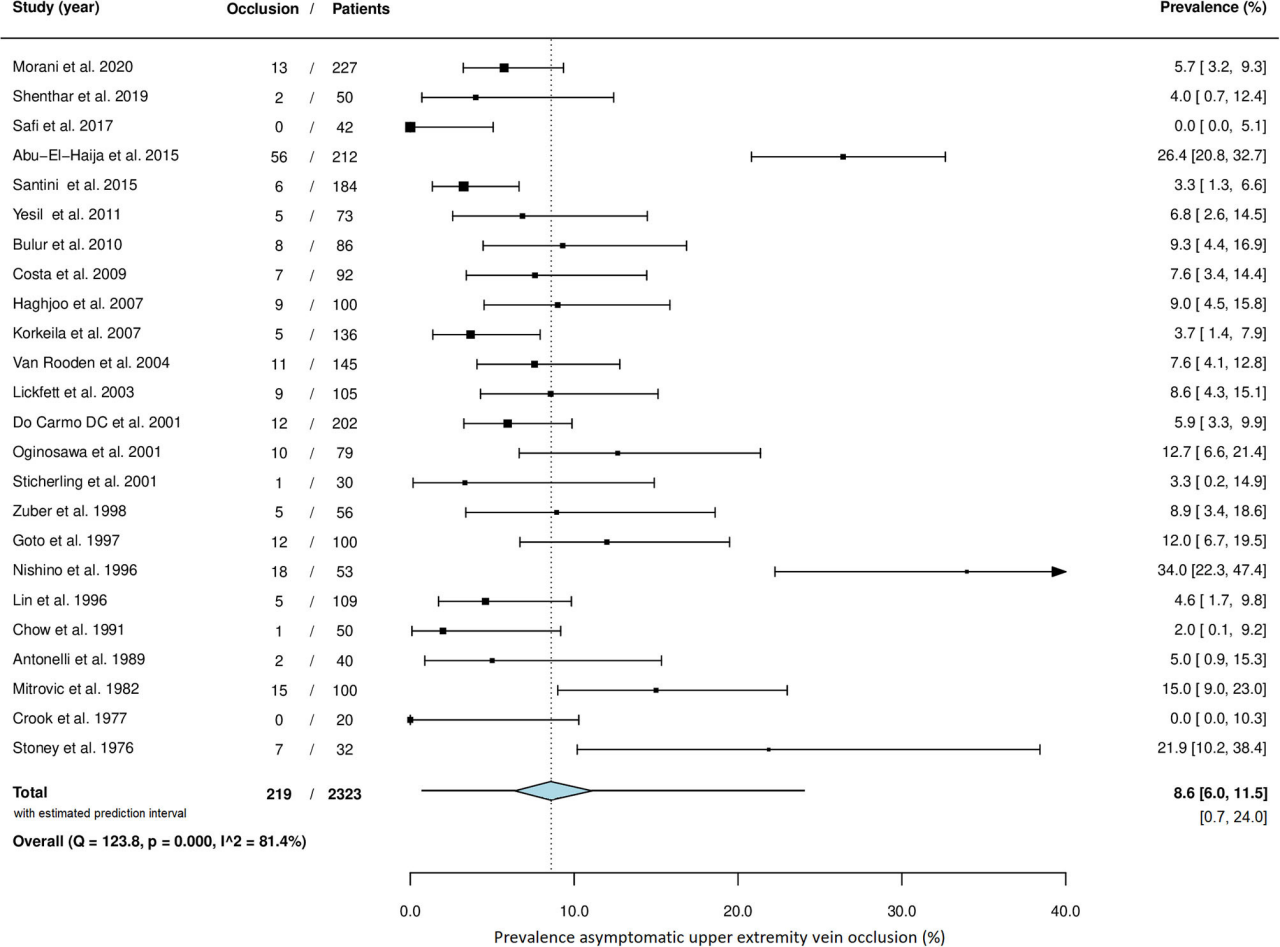
Meta-analysis of the prevalence of asymptomatic upper extremity vein occlusion.

**Figure 5:**
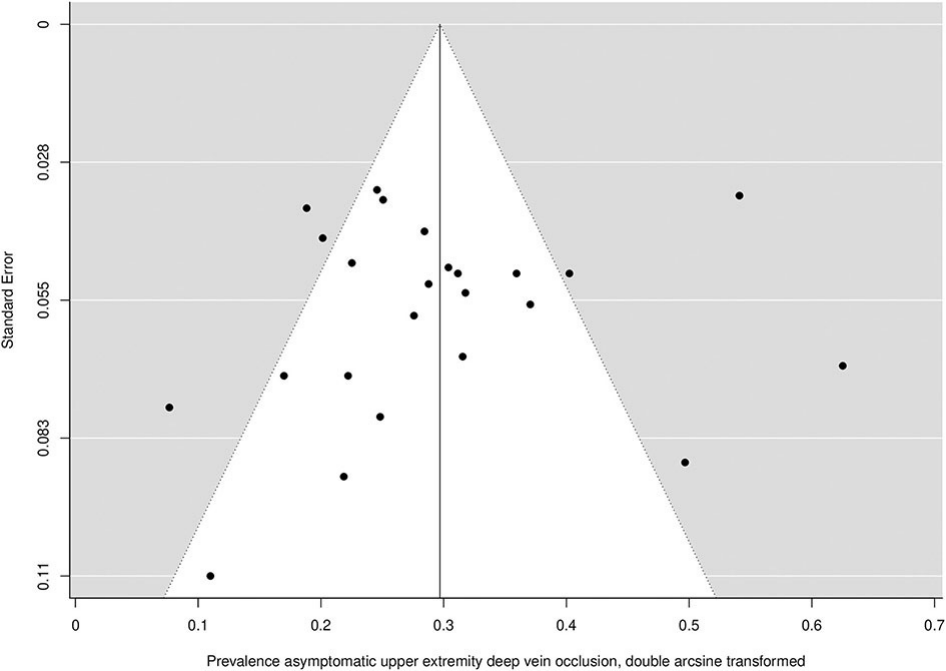
Funnel plot of the included studies for the prevalence of asymptomatic vein occlusion.

Asymptomatic upper extremity vein occlusion was present in 219 of the 2,323 patients. The prevalence ranged from 0.0 to 34.0% across studies. The overall pooled prevalence of asymptomatic vein occlusion is 8.6% (95% CI 6.0–11.5) with high heterogeneity among studies (I^2^ = 81.4%; *P* = <0.001) (Figure 4). Both Egger's test (*P* = 0.494) and the funnel plot (Figure 5) did not suggest the presence of publication bias.

Sensitivity analysis for asymptomatic vein occlusion (Table 2) showed a pooled prevalence of 6.5% (4.5–8.8) for prospective studies, 9.3% (3.6–17.1) for retrospective, and 10.3% (0.9–26.7) for cross-sectional studies. A pooled prevalence of 7.8% (4.7–11.4) was found in studies with low risk of bias. Combining those studies considered to be at low and moderate risk of bias resulted in a prevalence of 8.0% (5.2–11.3). The pooled prevalence of studies obtaining venogram of each included patient was 8.1% (5.2–11.6); this was 6.4% (3.8–9.6) for studies which did not.

**Table 2:** Subgroup analysis for the prevalence of asymptomatic vein occlusion.

**Subgroups**	**Studies (*n*)**	**Cases (*n*)**	**Patients (*n*)**	**Pooled prevalence % (95% CI)**	**Heterogeneity**	
					**I^**2**^**	**Q test's *P***
**Overall**						
	24	219	2,323	8.6 (6.0–11.5)	81.4	<0.001
**World part**						
Europe	8	64	973	6.1 (3.7–9.0)	60.4	0.013
Asia	6	48	441	9.8 (3.6–18.3)	84.3	<0.001
Middle East	5	24	341	5.9 (2.6–10.2)	49.6	0.094
USA	3	64	274	16.9 (5.2–33.2)	82.0	0.004
Brazil	2	19	294	6.1 (3.5–9.3)	0.0	0.555
**Design**						
Prospective	12	86	1,236	6.5 (4.5–8.8)	49.3	0.027
Retrospective	7	94	755	9.3 (3.6–17.1)	89.1	<0.001
Cross-sectional	4	34	223	10.3 (0.9–26.7)	89.3	<0.001
**Risk of bias**						
Low	15	157	1,631	7.8 (4.7–11.4)	82.5	<0.001
Low + moderate	22	202	2,065	8.0 (5.2–11.3)	82.7	<0.001
**Cardiac device**						
Pacemaker only	14	102	1,050	9.1 (5.8–12.9)	72.4	<0.001
ICD only	2	10	135	7.3 (3.3–12.4)	0.0	0.407
**Venography in all patients**						
Yes	21	198	2,013	8.1 (5.2–11.6)	83.5	<0.001
No	3	21	310	6.4 (3.8–9.6)	0.0	0.481
**Patients with venous anomalies excluded**						
Yes	3	13	237	3.6 (0.2–9.6)	65.1	0.057
No or not stated	21	206	2,086	8.7 (5.8–12.0)	82.3	<0.001

*CI, confidence interval*.

Meta-regression analyses showed no effect for publication year, age, sex, proportion of patients on anticoagulation therapy, mean number of leads per patient and mean follow-up duration on the prevalence of asymptomatic vein occlusion (Table 3).

**Table 3:** Univariate meta-regression for UEDVT and asymptomatic vein occlusion.

**Covariate**	**Populations (*n*)**	**β-coefficient**	**SE**	***p-*value**
**UEDVT**				
Publication year	21	−0.002	0.002	0.292
Age (μ)	21	0.002	0.002	0.389
Male sex (%)	19	−0.001	0.001	0.210
Anticoagulation (%)	10	0.000	0.001	0.981
No. of leads (μ)	18	0.066	0.022	*0.002*
**Asymptomatic vein occlusion**				
Publication year	24	−0.002	0.002	0.331
Age (μ)	21	0.005	0.006	0.387
Male sex (%)	22	−0.002	0.002	0.492
Anticoagulation (%)	13	0.000	0.002	0.880
No. of leads (μ)	19	0.016	0.075	0.831
Follow-up time (μ)	22	0.010	0.010	0.280

*SE, standard error; Italics indicates significance*.

## Discussion

The incidence rate of symptomatic UEDVT after the first 2 months following lead implantation ranged from 0.0 to 10.0 per 100 PY across individual studies and averaged ~0.9 UEDVT per 100 PY in the pooled analysis.

We report a substantial higher incidence of UEDVT among patients with transvenous leads when compared to data from the general population. An estimated incidence of 0.0036 primary and secondary UEDVT per 100 PY was found in the population of Malmö (Sweden) (62), whereas, an incidence of 0.025 UEDVT per 100 PY was reported in a French population aged between 60 and 74 years (63). The 35 to 244-fold higher incidence rate in patients with transvenous leads implies that the presence of these leads is an evident risk factor for symptomatic UEDVT.

The incidence we report underlines the difference in epidemiology between thrombotic complications immediately after lead implantation and those occurring after the initial postoperative period, suggesting a different etiology. Our results show that UEDVT is not reserved to the first months postoperatively but may occur up to multiple years after transvenous lead implantation. Given the large spread in timing of occurrence and low incidence, we do not advise routine screening on UEDVT in patients with transvenous leads.

It is unclear whether patients with transvenous leads warrant prophylactic anticoagulation for primary prevention of UEDVT. Our results do not provide guidance on the use of thromboprophylaxis postoperatively after lead implantation. After the initial postoperative months, the increased risk of major bleedings with direct oral anticoagulants (2.0–3.9 per 100 PY) and vitamin K antagonists (3.6 per 100 PY) as established in other populations, does not seem to outweigh any decrease in the risk of a first UEDVT (0.9 per 100 PY) in patients with transvenous leads (64).

When a first UEDVT occurs, transvenous leads should be considered a major provoking risk factor since the risk of UEDVT increases more than 10-fold after lead implantation (16). Further research is needed to provide insight into the association between leads and UEDVT over time to elucidate whether transvenous leads should be regarded a transient or persistent risk factor. An answer to this question would provide clarity as to whether anticoagulation may be stopped after the initial 3 months of treatment for a first VTE or extended anticoagulant treatment should be considered.

Significantly different UEDVT incidence rates were present in some subgroups compared to the overall pooled incidence. The lower incidence of UEDVT among retrospective studies compared to prospective studies may be explained by underreport of cases, especially with increasing follow-up duration. The pooled incidence for studies with only ICD patients was based on merely two studies and should therefore be interpreted with caution. In addition, the incidence of VTE is suggested to be generally lower in Asian compared to Western populations which might have resulted in a lower incidence rate among Asian studies in our analysis as well (65).

Furthermore, the results of our meta-regression analysis show that a higher number of transvenous leads is related to the occurrence of UEDVT, which is in line with earlier studies (38, 39, 43, 44, 54). A higher number of leads implies a larger total diameter of foreign intravascular material and may induce an increasingly thrombogenic environment. This is in line with the proposed pathophysiology of thrombosis around transvenous leads which includes lead endothelialisation, endovascular damage and venous stasis (4). A clinical application might be that dysfunctional transvenous leads–often let abandoned to avoid acute complications of extraction–are extracted in patients with a high thrombosis risk and low bleeding risk to prevent UEDVT in the long term.

Whereas, UEDVT could virtually affect all patients with transvenous leads, asymptomatic upper extremity vein occlusion is only relevant to those admitted for lead revision. The pooled analysis showed asymptomatic upper extremity vein occlusion to be present in 8.6% of the patients after transvenous lead placement. A previous comprehensive review reported a comparable prevalence of 8.3% of asymptomatic vein occlusion across seven studies; all of them, except for one, included in the current analysis as well (9). A more recent review stated that asymptomatic vein occlusion is present in 2–25% of the patients after lead placement, which lies entirely within the range of 0 to 34% on which we report (4). Given a 1-year reintervention rate of 4.2% after pacemaker implantation and 6.3% after ICD or CRT implantation as found in the UK national audit 2017, upper extremity vein occlusion frequently causes clinical difficulties (66).

### Strengths and Limitations

The results of the present analysis apply to all patients with transvenous leads; patients with both active as well as abandoned leads, patients from different continents and patients with all types of cardiac devices were included. In addition, we used well-defined outcomes which are clinically relevant to both patient and physician.

However, several limitations should be addressed when interpreting the results of the current meta-analysis, especially regarding UEDVT incidence. First, the incidence rate of UEDVT might have been underestimated as the studies did not use a uniform definition. Second, only 42 out of 72 UEDVT could be confirmed to have occurred at ≥2 months postoperatively. As a result, the reported UEDVT incidence may be an overestimation of the actual incidence. Third, only three studies were of low risk of bias which implies that the overall pooled incidence was derived from studies of poor quality predominantly. Lastly, a substantial amount of heterogeneity was present among the studies. Methodological heterogeneity arose from difference in study design; clinical heterogeneity followed from inclusion of both studies assessing patients with functional and non-functional (abandoned) leads.

Regarding asymptomatic vein occlusion, we determined the prevalence of total vein occlusion only. Sub-occlusive upper extremity vein stenoses may occur even more frequent but are clinically less relevant in case of lead revision. In addition, heterogeneity in the reported categories of venous obstruction across studies hampered pooled analyses.

In the meta-analysis of both outcomes, substantial statistical heterogeneity was present, and could not be explained sufficiently by sensitivity and meta-regression analyses.

## Conclusions

The incidence of symptomatic UEDVT in patients with transvenous leads is 0.9 per 100 PY after the first 2 months following lead implantation. Given the much lower incidence of UEDVT in the general population, the presence of transvenous leads must be considered an important risk factor for UEDVT. In addition, the presence of more transvenous leads was identified as a potential risk factor for UEDVT. The prevalence of asymptomatic upper extremity vein occlusion after transvenous lead implantation is 8.6%. Although, this condition will remain subclinical in most cases, vein occlusion will complicate any future lead addition. Future research should assess clinically relevant outcomes–e.g., symptomatic UEDVT and asymptomatic vein occlusion–and focus on risk factors for thrombotic complications and the role of prophylactic anticoagulation therapy in patients with transvenous leads. In order to gain more high-quality data on the epidemiology of thrombotic complications after lead implantation, we advise to conduct dedicated prospective studies and we call registries to record UEDVT as a complication of lead implantation with a focus on time between implantation and diagnosis.

## Data Availability Statement

The original contributions presented in the study are included in the article/Supplementary Material, further inquiries can be directed to the corresponding author/s.

## Author Contributions

DD and MW: conceived and designed the analysis, collected data, performed the analysis, and wrote the paper. MN and JW: conceived and designed the analysis and substantial contributions to the text of the paper. AT: substantial contributions to the text of the paper and feedback on the content of the presented data. All authors contributed to manuscript revision, read, and approved the submitted version.

## Conflict of Interest

The authors declare that the research was conducted in the absence of any commercial or financial relationships that could be construed as a potential conflict of interest.

## Publisher's Note

All claims expressed in this article are solely those of the authors and do not necessarily represent those of their affiliated organizations, or those of the publisher, the editors and the reviewers. Any product that may be evaluated in this article, or claim that may be made by its manufacturer, is not guaranteed or endorsed by the publisher.

## Abbreviations

CRT: Cardiac Resynchronization Therapy
ICD: Implantable Cardioverter-Defibrillator
PM: Pacemaker
PY: Patient Years
UEDVT: Upper Extremity Deep Vein Thrombosis.
