# Supplementary material for: Upper Extremity Deep Vein Thrombosis and Asymptomatic Vein Occlusion in Patients With Transvenous Leads: A Systematic Review and Meta-Analysis
Source: Front Cardiovasc Med. 2021 Aug 18;8:698336. doi: 10.3389/fcvm.2021.698336 (PMC8416492; doi:10.3389/fcvm.2021.698336)
Supplement: Supplementary file 1 [file Data_Sheet_1.docx]

# Supplementary data

## Supplement 1: Search term

### Pubmed

(thrombosis[tiab] OR thromboses[tiab] OR thrombus[tiab] OR thrombi[tiab] OR blood clot*[tiab] OR thrombotic[tiab] OR thromboemboli*[tiab] OR thrombo emboli*[tiab] OR DVT[tiab] OR UEDVT[tiab] OR VTE[tiab] OR ((vein[tiab] OR venous[tiab]) AND (occlusion*[tiab] OR stenosis[tiab] OR obstruction*[tiab] OR patency[tiab])) OR embolism and thrombosis[MeSH Terms] OR embolism*[tiab] OR embolization*[tiab] OR embolisation*[tiab] OR embolized[tiab] OR embolised[tiab] OR embolizing[tiab] OR embolising[tiab] OR embolus[tiab] OR PE[tiab] OR ((lung[tiab] OR pulmon*[tiab]) AND (embol*[tiab] OR thrombus[tiab] OR thrombosis[tiab] OR thromboses[tiab] OR thrombi[tiab] OR clot*[tiab])) OR superior vena cava syndrome*[tiab] OR superior vena cava obstruction*[tiab] OR superior vena cava occlusion*[tiab] OR superior vena cava stenosis[tiab] OR superior vena cava thromb*[tiab] OR superior vena caval thromb*[tiab] OR superior vena caval obstruction*[tiab] OR superior vena caval occlusion*[tiab] OR superior vena caval stenosis[tiab] OR svc syndrome[tiab] OR svc obstruction*[tiab] OR svc stenosis[tiab] OR svc occlusion*[tiab] OR Superior vena cava syndrome[MeSH Terms] OR Vena cava, superior[MeSH Terms]) **AND** (cardiac device*[tiab] OR intracardiac device*[tiab] OR cardiac rhythm device*[tiab] OR cardiac implantable device*[tiab] OR cardiac implantable electronic*[tiab] OR cardiovascular device*[tiab] OR pacemaker*[tiab] OR implantable cardioverter*[tiab] OR implanted cardioverter*[tiab] OR implantable defibrillator*[tiab] OR implanted defibrillator*[tiab] OR cardiac resynchronization[tiab] OR cardiac resynchronisation[tiab] OR AICD[tiab] OR ((transvenous[tiab] OR transvenous[tiab] OR endovenous[tiab] OR intravascular[tiab] OR transvascular[tiab] OR endovascular[tiab] OR intracardia*[tiab] OR transcardia*[tiab] OR endocardia*[tiab] OR cardia*)[tiab] AND (lead[tiab] OR leads[tiab] OR wire*[tiab] OR electrode*[tiab] OR pacing[tiab])) OR Pacemaker, Artificial[MeSH Terms] OR Cardiac pacing, artificial[MeSH Terms] OR Electrodes, implanted[MeSH Terms])

### Embase

(‘thrombosis':ti,ab,kw OR 'thromboses':ti,ab,kw OR 'thrombus':ti,ab,kw OR 'thrombi':ti,ab,kw OR 'blood clot*':ti,ab,kw OR 'thrombotic':ti,ab,kw OR 'thromboemboli*':ti,ab,kw OR 'thrombo emboli*':ti,ab,kw OR 'dvt':ti,ab,kw OR 'uedvt':ti,ab,kw OR 'vte':ti,ab,kw OR (((venous OR vein) NEXT/1 (stenosis OR occlusion* OR obstruction* OR patency)):ti,ab,kw) OR 'embolism*':ti,ab,kw OR 'emboli?ation*':ti,ab,kw OR 'emboli?ed':ti,ab,kw OR 'emboli?ing':ti,ab,kw OR 'embolus':ti,ab,kw OR 'pe':ti,ab,kw OR (((lung OR pulmon*) NEXT/1 (embol* OR thrombus OR thrombosis OR thromboses OR thrombi OR clot*)):ti,ab,kw) OR 'lung embolism'/exp OR ‘vein embolism'/exp OR ‘intracardiac thrombosis'/exp OR ‘postoperative thrombosis'/exp OR ‘thrombus'/exp OR ‘vein thrombosis'/exp OR ‘upper extremity deep vein thrombosis'/exp OR ‘deep vein thrombosis'/exp OR 'superior vena cava* syndrome*':ti,ab,kw OR 'superior vena cava* obstruction*':ti,ab,kw OR 'superior vena cava* occlusion*':ti,ab,kw OR 'superior vena cava* thromb*':ti,ab,kw OR 'svc syndrome':ti,ab,kw OR 'svc obstruction*':ti,ab,kw OR 'svc stenosis':ti,ab,kw OR 'svc occlusion*':ti,ab,kw OR 'superior cava vein obstruction'/exp) **AND** ('cardiac device*':ti,ab,kw OR 'intracardiac device*':ti,ab,kw OR 'cardiac rhythm device*':ti,ab,kw OR 'cardiac implantable device*':ti,ab,kw OR 'cardiac implantable electronic*':ti,ab,kw OR 'cardiovascular device*':ti,ab,kw OR 'pacemaker*':ti,ab,kw OR 'implantable cardioverter*':ti,ab,kw OR 'implanted cardioverter*':ti,ab,kw OR 'implantable defibrillator*':ti,ab,kw OR 'implanted defibrillator*':ti,ab,kw OR 'cardiac resynchroni?ation':ti,ab,kw OR 'aicd':ti,ab,kw OR (((transvenous OR transvenous OR endovenous OR intravascular OR transvascular OR endovascular OR intracardia* OR transcardia* OR endocardia* OR cardia*) NEXT/1 (lead OR leads OR wire* OR electrode* OR pacing)):ti,ab,kw) OR 'cardiac electrode'/exp OR ‘artificial heart pacemaker'/exp OR ‘cardiac implantable electronic device'/exp OR ‘defibrillator'/exp OR ‘pacemaker accessory'/exp) **AND** ([dutch]/lim OR [english]/lim OR [french]/lim OR [german]/lim)

## Supplement 2: Data extraction form

| Date of extraction |  |
| --- | --- |
| Extractor ID |  |
| First author |  |
| Year of publication |  |
| Contact details |  |
| Country of origin |  |
| Study design |  |
| Study aim |  |
| Patient in/exclusion criteria |  |
| Sample size |  |
| Follow-up duration |  |
| Type of population | FU after implantation / abandoned leads / prior to intervention |
| Population characteristics |  |
| Age |  |
| Sex |  |
| Predisposing factors |  |
| Anticoagulant/antiplatelet use |  |
| Comorbidities |  |
| Indication for intracardiac device |  |
| Type of cardiac device |  |
| Number of leads |  |
| Definition of outcome(s) |  |
| Assessment of outcome(s) |  |
| Incidence UEDVT |  |
| Follow-up time at UEDVT(s) |  |
| Prevalence asymptomatic deep vein occlusion |  |

|  | | Supplement 3: Risk of bias assessment of studies included in the meta-analysis | **Was the study's target population a close representation of the national population?** | | **Was random selection used to select the sample?**  **Was the sampling frame a true/close representation of the target population?** | | | **Were data collected directly from subjects?**  **Was the likelihood of non-response bias minimal?** | | | **Was an acceptable case definition used?**  **Was the study instrument that measured the parameter of interest valid?** | | **Was the same mode of data collection used for all subjects?** | | | **Were the numerator and denominator appropriate?** | |  | | **Risk of Bias** |  |
| --- | --- | --- | --- | --- | --- | --- | --- | --- | --- | --- | --- | --- | --- | --- | --- | --- | --- | --- | --- | --- | --- |
| **Author (year)** |  | |  | |  | | |  | | |  | | ? | | |  | | **Summary score Risk of Bias** | |  |  |
| **Symptomatic UEDVT and Asymptomatic occlusion** |  | |  | |  | | |  | | |  | |  | | |  | |  | |  |  |
| Shenthar et al. (2019) | Sym | |  |  | |  |  | |  |  | |  | |  |  | | 2 | | Moderate | |  |
|  | Asym | |  |  |  |  |  |  |  |  | |  |  |  |  |  | 1 | | Low | |  |
| Safi et al. (2017) | Sym | | NS |  | |  |  | |  |  | |  | |  |  | | 4 | | High | |  |
|  | Asym | |  |  |  |  |  |  |  |  | |  |  |  |  |  | 3 | | Moderate | |  |
| Bulur et al. (2010) | Sym | |  |  | | NS | NS | |  |  | |  | |  |  | | 4 | | High | |  |
|  | Asym | |  |  |  |  |  |  |  |  | |  |  |  |  |  | 3 | | Moderate | |  |
| Costa et al. (2009) | Sym | |  |  | |  |  | |  |  | |  | |  |  | | 2 | | Moderate | |  |
|  | Asym | |  |  |  |  |  |  |  |  | |  |  |  |  |  | 2 | | Moderate | |  |
| Korkeila et al. (2007) | Sym | |  |  | |  |  | |  |  | |  | |  |  | | 1 | | Low | |  |
|  | Asym | |  |  |  |  |  |  |  |  | |  |  |  |  |  | 1 | | Low | |  |
| Van Rooden et al. (2004) | Sym | |  |  | |  |  | |  |  | |  | |  |  | | 1 | | Low | |  |
|  | Asym | |  |  |  |  |  |  |  |  |  |  |  |  |  |  | 1 | | Low | |  |
| Do Carmo Da Costa et al. (2001) | Sym | |  |  | | NS | NS | |  |  | |  | |  |  | | 5 | | High | |  |
|  | Asym | |  |  |  |  |  |  |  |  | |  |  |  |  |  | 4 | | High | |  |
| Oginosawa et al. (2001) | Sym | |  |  | |  |  | |  |  | |  | |  |  | | 2 | | Moderate | |  |
|  | Asym | |  |  |  |  |  |  |  |  | |  |  |  |  |  | 1 | | Low | |  |
| Lin et al. (1996) | Sym | |  |  | |  |  | |  |  | |  | |  |  | | 2 | | Moderate | |  |
|  | Asym | |  |  |  |  |  |  |  |  | |  |  |  |  |  | 2 | | Moderate | |  |
| Antonelli et al. (1989) | Sym | |  |  | |  |  | |  |  | |  | |  |  | | 2 | | Moderate | |  |
|  | Asym | |  |  |  |  |  |  |  |  | |  |  |  |  |  | 1 | | Low | |  |
| **Symptomatic UEDVT** |  | |  |  | |  |  | |  |  | |  | |  |  | |  | |  | |  |
| Holzhauser et al. (2018) |  | |  |  | |  |  | |  |  | |  | |  |  | | 1 | | Low | |  |
| Miyzaki et al. (2013) |  | |  |  | |  |  | |  |  | | NS | |  |  | | 3 | | Moderate | |  |
| Bode et al. (2012) |  | |  |  | |  |  | |  |  | |  | |  |  | | 2 | | Moderate | |  |
| Lelakowski et al. (2011) |  | |  |  | |  |  | |  |  | |  | |  |  | | 2 | | Moderate | |  |
| Glikson et al. (2009) |  | |  |  | | NS | NS | |  |  | | NS | | NS |  | | 7 | | High | |  |
| Juszkat et al. (2006) |  | |  |  | | NS | NS | |  |  | |  | |  |  | | 4 | | High | |  |
| Kar et al. (2000) |  | |  |  | | NS | NS | |  |  | |  | |  |  | | 4 | | High | |  |
| Levy et al. (2000) |  | |  |  | | NS | NS | |  |  | | NS | |  |  | | 6 | | High | |  |
| De Cock et al. (1999) |  | |  |  | | NS | NS | |  |  | | NS | |  |  | | 4 | | High | |  |
| Zerbe et al. (1985) |  | |  |  | | NS | NS | |  |  | |  | |  |  | | 6 | | High | |  |
| **Asymptomatic occlusion** |  | |  |  | |  |  | |  |  | |  | |  |  | |  | |  | |  |
| Morani et al. (2020) |  | | NS |  | |  |  | |  |  | |  | |  |  | | 1 | | Low | |  |
| Abu-El-Haija et al. (2015) |  | | NS |  | |  |  | |  |  | |  | |  |  | | 1 | | Low | |  |
| Santini et al. (2015) |  | |  |  | |  |  | |  |  | |  | |  |  | | 0 | | Low | |  |
| Yesil et al. (2011) |  | |  |  | |  |  | |  |  | |  | |  |  | | 1 | | Low | |  |
| Haghjoo et al. (2007) |  | |  |  | |  |  | |  |  | |  | |  |  | | 0 | | Low | |  |
| Lickfett et al. (2003) |  | |  |  | |  |  | |  |  | |  | |  |  | | 1 | | Low | |  |
| Stichterling et al. (2001) |  | |  |  | |  |  | |  |  | |  | |  |  | | 1 | | Low | |  |
| Zuber et al. (1998) |  | |  |  | | NS | NS | |  |  | |  | |  |  | | 4 | | High | |  |
| Goto et al. (1997) |  | |  |  | |  |  | |  |  | |  | |  |  | | 1 | | Low | |  |
| Nishino et al. (1996) |  | |  |  | |  |  | |  |  | |  | |  |  | | 2 | | Moderate | |  |
| Chow et al. (1991) |  | |  |  | |  |  | |  |  | |  | |  |  | | 1 | | Low | |  |
| Mitrovic et al. (1983) |  | |  |  | |  |  | |  |  | |  | |  |  | | 1 | | Low | |  |
| Crook et al. (1977) |  | |  |  | |  |  | |  |  | |  | |  |  | | 2 | | Moderate | |  |
| Stoney et al. (1976) |  | |  |  | |  | NS | |  |  | |  | |  |  | | 3 | | Moderate | |  |

Green indicates Yes; Red, No; NS = not stated.

Sym = symptomatic UEDVT; Asym = asymptomatic venous occlusion.

Cut-off values: 0-1 = low risk of bias; 2-3 = moderate risk of bias; 4-9 = high risk of bias.

| **Supplement 4.** Demographics and characteristics of included studies for the incidence rate of UEDVT | | | | | | | | |  |  |  |  |  |
| --- | --- | --- | --- | --- | --- | --- | --- | --- | --- | --- | --- | --- | --- |
| **First author (publication year)** | **Country** | **Study design** | **Sample size (n)** | **Age**  **(y)** | **Male**  **(%)** | **Type of population** | **PM/ICD/CRT (%)** | **Anticoagulat**  **ion (%)** | | **Risk of Bias** | **Follow-up (year)** | **UEDVT**  **per 100 PY** | |
| Shenthar et al. (2019)^49^ | India | Prospective | 50 | 55.9±14.4 | 56 | FU after implantation | 100/0/0 | 14 | | Moderate | 4.3±4.1 | 0.0 | |
| Holzhauser et al. (2018)^23^ | USA | Retrospective | 37 | 57.8±7.4 | 70 | Abandoned leads | 0/32/68 | NA | | Low | 6.4±4.0 | 1.7 | |
| Safi et al. (2017)^50^ | Iran | Prospective | 42 | 59.7±12.3 | 69 | FU after implantation | 36/43/21 | 0 | | High | 0.4 | 10.0 | |
| Miyazaki et al. (2013)^24^ | Japan | Retrospective | 82 | 53.5±13.6 | 87 | FU after implantation | 0/100/0 | NA | | Moderate | 6.0±2.8° | 0.2 | |
| Bode et al. (2012)^29^ | Germany | Retrospective | 903 | 64.4±11.4 | 81 | FU after implantation | 0/78/22 | NA | | Moderate | 4.1±3.2 | 0.2* | |
| Lelakowski et al. (2011)^30^ | Poland | Prospective | 81 | 71.1±7.6 | 62 | FU after implantation | 100/0/0 | 36 | | Moderate | 1.6±0.0 | 2.6 | |
| Bulur et al. (2010)^53^ | Turkey | Prospective | 86 | 61±13 | 66 | FU after implantation | 100/47/0 | 33 | | High | 1.7±1.4 | 0.0 | |
| Costa et al. (2009)^54^ | Brazil | Prospective | 101 | 61.0±11.7 | 43 | FU after implantation | 46/10/43 | 49 | | Moderate | 0.5±0.0 | 0.0 | |
| Glikson et al. (2009)^31^ | USA | Prospective | 78 | 63±14 | 81 | Abandoned leads | 0/100/0 | NA | | High | 5.4±3.1 | 0.0 | |
| Korkeila et al. (2007)^55^ | Finland | Prospective | 136 | 66.6±12.4 | 62 | FU after implantation | 86/14/0 | 46 | | Low | 0.5±0.0 | 1.5* | |
| Juszkat et al. (2006)^32^ | Poland | Retrospective | 106 | 45.6 | 43 | FU after implantation | 100/0/0 | 14 | | High | 2.9 | 0.7 | |
| Van Rooden et al. (2004)^56^ | The Netherlands | Prospective | 145 | 62.4 | 72 | FU after implantation | 48/52/0 | 59 | | Low | 1±0.0 | 1.7 | |
| Do Carmo DC et al. (2001)^57^ | Brazil | Prospective | 202 | 64.3±16.6 | 51 | FU after implantation | 100/0/0 | NA | | High | 0.5±0.0 | 5.9* | |
| Oginosawa et al. (2001)^58^ | Japan | Prospective | 79 | 71.9±9.0 | 46 | FU after implantation | 100/0/0 | NA | | Moderate | 3.7±0.5 | 0.0 | |
| Kar et al. (2000)^34^ | India | Prospective | 6256 | 46±7.3 | 93 | FU after implantation | 100/0/0 | NA | | High | 4.7±1.2 | 0.09 | |
| Levy et al. (2000)^33^ | United Kingdom | Prospective | 21 | 63 | 57 | FU after implantation | 100/0/0 | NA | | High | 0.8±0.4 | 7.7 | |
| De Cock et al. (1999)^35^_controls_ | The Netherlands | Prospective | 48 | 62±8 | NA | FU after implantation | 100/0/0 | 14.6 | | High | 7.4±2 | 2.0* | |
| De Cock et al. (1999)^35^ _abandoned_ | The Netherlands | Prospective | 48 | 62±9 | NA | Abandoned leads | NA | 6.3 | | High | 7.4±2 | 2.3* | |
| Lin et al. (1996)^59^ | Taiwan | Unclear | 109 | 72±10 | 49 | FU after implantation | 100/0/0 | NA | | Moderate | 3.6±2.6 | 0.0 | |
| Antonelli et al. (1989)^60^ | Israel | Prospective | 40 | 68±18 | 60 | FU after implantation | 100/0/0 | NA | | Moderate | 1.4 | 2.1 | |
| Zerbe et al. (1985)^36^ | Poland | Retrospective | 21 | 64.9 | 81 | Abandoned leads | 100/0/0 | NA | | High | 4.4 | 2.3 | |
| *Alvarez et al. (2018)*^25^ | USA | Retrospective | 49 | 59.9±10.7° | NA | Abandoned leads | 0/35/65 | NA | | Low | NA | 2/49 | |
| *Rahbi et al. (2014)*^26^ | United Kingdom | Prospective | 151 | 74.3±10.0° | 83 | FU after implantation | 0/24/76 | NA | | Moderate | 1.0 (med) | 0/151 | |
| *Bohm et al. (2001)*^27^ | Hungary | Retrospective | 60 | 57.4 | 50 | Abandoned leads | 100/0/0 | NA | | High | NA | 2/60* | |
| *Williams et al.* *(1978)*^28^ | USA | Retrospective | 212 | NA | NA | FU after implantation | 100/0/0 | NA | | Moderate | NA | 2/212 | |
| *Crook et al. (1977)*^51^ | United Kingdom | Cross-sectional | 125 | NA | NA | FU after implantation | 100/0/0 | NA | | Moderate | 0.3-5 (range) | 1/125 | |
| *Stoney et al. (1976)*^52^ | USA | Cross-sectional | 32 | NA | NA | Prior to intervention | 100/0/0 | NA | | High | >1.5 | 1/32 | |
| Continuous variables are presented as µ(±SD) or otherwise specified.  NA = not assessed; *Italics* indicates: not included in meta-analysis, incidence rate cannot be calculated (prevalence is given); PY = person years of follow-up; FU = follow-up; med = median.  °estimated from median (IQR, range)  *no. of cases within 2 months postoperatively unknown | | | | | | | | | | | | |  |

| **Supplement 5.** Demographics and characteristics of included studies for the prevalence of asymptomatic upper extremity vein occlusion | | | | | | | | | | | |  |  |
| --- | --- | --- | --- | --- | --- | --- | --- | --- | --- | --- | --- | --- | --- |
| **First author (publication year)** | **Country** | **Study design** | **Sample size (n)** | **Age**  **(y)** | **Male**  **(%)** | **Type of population** | **PM/ICD/CRT (%)** | **Anticoagu**  **lation (%)** | **Initial diagnostic** | **Risk of Bias** | **Follow-up (year)** | | **Prevalence occlusion (%)** |
| Morani et al. (2020)^37^ | Italy | Prospective | 227 | 72±9 | 80 | Prior to intervention | 45/33/23 | NA | Venography | Low | 5.6±3.9 | | 5.7 |
| Shenthar et al. (2019)^49^ | India | Prospective | 50 | 55.9±14.4 | 56 | FU after implantation | 100/0/0 | 14 | Venography | Low | 4.3±4.1 | | 4.0 |
| Safi et al. (2017)^50^ | Iran | Prospective | 42 | 59.7±12.3 | 69 | FU after implantation | 36/43/21 | 0 | Venography | Moderate | 0.4 | | 0.0 |
| Abu-El-Haija et al. (2015)^38^ | USA | Retrospective | 212 | 69±14.3 | 64 | Prior to intervention | NA/NA/NA | 38 | Venography | Low | 6.2 | | 26.4 |
| Santini et al. (2015)^41^ | Italy | Retrospective | 184 | 71.6±9.7° | 67 | FU after implantation | 72/22/7 | 26 | Venography | Low | 6.3±5.2° | | 3.3 |
| Yesil et al. (2011)^42^ | Turkey | Retrospective | 73 | 68.8±13.5 | 44 | Prior to intervention | 100/0/0 | 6 | Venography | Low | 10.8±5.7 | | 6.8 |
| Bulur et al. (2010)^53^ | Turkey | Prospective | 86 | 61±13 | 66 | FU after implantation | 100/47/0 | 33 | Venography | Moderate | 1.7±1.4 | | 9.3 |
| Costa et al. (2009)^54^ | Brazil | Prospective | 101 | 61.0±11.7 | 43 | FU after implantation | 46/10/43 | 49 | Venography | Moderate | 0.5±0.0 | | 7.6 |
| Haghjoo et al. (2007)^43^ | Iran | Retrospective | 100 | 62.8±19.4 | 53 | Prior to intervention | 83/17/0 | 11 | Venography | Low | 8.1±4.5 | | 9.0 |
| Korkeila et al. (2007)^55^ | Finland | Prospective | 136 | 66.6±12.4 | 62 | FU after implantation | 86/14/0 | 46 | Venography | Low | 0.5±0.0 | | 3.7 |
| Van Rooden et al. (2004)^56^ | The Netherlands | Prospective | 145 | 62.4 | 72 | FU after implantation | 48/52/0 | 59 | Ultrasound | Low | 1±0.0 | | 7.6 |
| Lickfett et al. (2003)^44^ | Germany | Prospective | 105 | NA | 83 | Prior to intervention | 0/100/0 | NA | Venography | Low | 3.9±1.0 | | 8.6 |
| Do Carmo DC et al. (2001)^57^ | Brazil | Prospective | 202 | 64.3±16.6 | 51 | FU after implantation | 100/0/0 | NA | Venography | High | 0.5±0.0 | | 5.9 |
| Oginosawa et al. (2001)^58^ | Japan | Prospective | 79 | 71.9±9.0 | 46 | FU after implantation | 100/0/0 | NA | Venography | Low | 3.7±0.5 | | 12.7 |
| Sticherling et al. (2001)^45^ | USA | Retrospective | 30 | 60±15 | 83 | Prior to intervention | 0/100/0 | 23 | Venography | Low | 3.9±1.8 | | 3.3 |
| Zuber et al. (1998)^46^ | Switzerland | Retrospective | 56 | 71.3±10.6 | 55 | FU after implantation | 100/0/0 | NA | Ultrasound | High | 3.5±3.8 | | 8.9 |
| Goto et al. (1997)^47^ | Japan | Retrospective | 100 | 70±9 | 54 | Prior to intervention | 100/0/0 | 15 | Venography | Low | 6.0±1.4 | | 12.0 |
| Nishino et al. (1996)^48^ | Japan | Cross-sectional | 53 | 68±10 | 55 | FU after implantation | 100/0/0 | 0 | Ultrasound | Moderate | 4 | | 34.0 |
| Lin et al. (1996)^59^ | Taiwan | Unclear | 109 | 72±10 | 49 | FU after implantation | 100/0/0 | NA | Ultrasound | Moderate | 3.6±2.6 | | 4.6 |
| Chow et al. (1991)^39^ | Hong Kong | Cross-sectional | 50 | 66 | 50 | FU after implantation | 100/0/0 | NA | Venography | Low | 4 | | 2.0 |
| Antonelli et al. (1989)^60^ | Israel | Prospective | 40 | 68±18 | 60 | FU after implantation | 100/0/0 | NA | Venography | Low | 1.4 | | 5.0 |
| Mitrovic et al. (1983)^40^ | Germany | Cross-sectional | 100 | 62 | 54 | FU after implantation | 100/0/0 | NA | Venography | Low | 3.7±0.9 | | 15.0 |
| Crook et al. (1977)^51^ | UK | Cross-sectional | 20 | 30-81(range) | NA | FU after implantation | 100/0/0 | NA | Venography | Moderate | NA | | 0.0 |
| Stoney et al. (1976)^52^ | USA | Prospective | 32 | NA | NA | Prior to intervention | 100/0/0 | NA | Venography | Moderate | >1.5 | | 21.9 |
| Continuous variables are presented as µ(±SD) or otherwise specified.  NA = not assessed; FU = follow-up.  °estimated from median (IQR, range) | | | | | | | | | | | | | |

| **Supplement 6.** Subgroup analysis for the incidence of symptomatic UEDVT without the two largest studies | | | | | | | |
| --- | --- | --- | --- | --- | --- | --- | --- |
| **Subgroups** | **Populations**  **(n)** | **Cases**  **(n)** | **PY**  **(n)** | **UEDVT per 100 PY**  **% (95%CI)** | **Heterogeneity** | | |
|  |  |  |  |  | **I^2^** | **Q test’s *P*** | |
| **Overall** |  |  |  |  |  |  | |
|  | 19 | 39 | 3698 | 1.2 (0.6 – 2.0) | 71.1 | <0.001 | |
| **Region** |  |  |  |  |  |  | |
| Europe | 8 | 26 | 1402 | 1.3 (0.7 – 2.1) | 0.0 | 0.579 | |
| Asia | 4 | 1 | 1337 | 0.0 (0.0 – 0.0) | 0.0 | 0.836 | |
| Middle East | 3 | 2 | 185 | 1.5 (0.0 – 8.0) | 65.3 | 0.056 | |
| USA | 2 | 4 | 659 | 0.1 (0.0 – 2.9) | 87.1 | 0.005 | |
| Brazil | 2 | 6 | 134 | 2.7 (0.0 – 11.0) | 65.2 | 0.090 | |
| **Design** |  |  |  |  |  |  | |
| Prospective | 14 | 30 | 2239 | 0.8 (0.1 – 2.0) | 72.9 | <0.001 | |
| Retrospective | 4 | 9 | 1086 | 0.4 (0.0 – 1.5) | 55.2 | 0.083 | |
| **Risk of bias** |  |  |  |  |  |  | |
| Low | 3 | 7 | 420 | 1.3 (0.3 – 2.8) | 0.0 | 0.994 | |
| Low + Moderate | 10 | 12 | 1953 | 0.2 (0.0 – 0.9) | 58.8 | 0.009 | |
| **Cardiac device** |  |  |  |  |  |  | |
| Pacemaker only | 11 | 22 | 1994 | 0.6 (0.0 – 1.8) | 74.4 | <0.001 | |
| ICD only | 2 | 1 | 886 | 0.0 (0.0 – 0.03) | 0.0 | 0.366 | |
| **Population** |  |  |  |  |  |  | |
| Follow-up after device implantation | 15 | 25 | 2615 | 0.5 (0.0 – 1.4) | 68.8 | <0.001 | |
| Abandoned leads | 4 | 14 | 1083 | 0.7 (0.0 – 2.9) | 81.9 | 0.001 | |
| **Timing of events** |  |  |  |  |  |  | |
| All UEDVT ≥2 months post-op | 14 | 17 | 2786 | 0.2 (0.0 – 0.8) | 63.3 | 0.001 | |
| no. UEDVT <2 months post-op unknown | 5 | 22 | 912 | 1.8 (0.8 – 3.2) | 16.3 | 0.311 | |
| **Patients with venous anomalies excluded** |  |  |  |  |  |  | |
| Yes | 4 | 6 | 452 | 1.1 (0.0 – 4.5) | 70.1 | 0.018 | |
| No or not stated | 15 | 33 | 3246 | 0.5 (0.0 – 1.3) | 72.9 | <0.001 | |
| PY = person years of follow-up; UEDVT = symptomatic upper extremity deep vein thrombosis; CI = confidence interval; post-op = postoperatively. | | | | | | |  |
